# Development of Trust in an Online Breast Cancer Forum: A Qualitative Study

**DOI:** 10.2196/jmir.7471

**Published:** 2017-05-23

**Authors:** Melanie Lovatt, Peter A Bath, Julie Ellis

**Affiliations:** ^1^ Faculty of Social Sciences University of Stirling Stirling United Kingdom; ^2^ Health Informatics Research Group Information School University of Sheffield Sheffield United Kingdom; ^3^ Department of Sociological Studies University of Sheffield Sheffield United Kingdom

**Keywords:** trust, online information sharing, breast cancer, online health communities, qualitative research

## Abstract

**Background:**

Online health forums provide peer support for a range of medical conditions including life-threatening and terminal illnesses. Trust is an important component of peer-to-peer support, although relatively little is known about how trust forms within online health forums.

**Objective:**

The aim of this paper is to examine how trust develops and influences sharing among users of an online breast cancer forum.

**Methods:**

An interpretive qualitative approach was adopted. Data were collected from forum posts from 135 threads on 9 boards on the UK charity, Breast Cancer Care (BCC). Semistructured interviews were conducted with 14 BCC forum users. Both datasets were analyzed thematically using Braun and Clarke’s approach and combined to triangulate analysis.

**Results:**

Trust operates in 3 dimensions, structural, relational, and temporal, and these intersect with each other and do not operate in isolation. The structural dimension relates to how the affordances and formal rules of the site affected trust. The relational dimension refers to how trust was necessarily experienced in interactions with other forum users: it emerged within relationships and was a social phenomenon. The temporal dimension relates to how trust changed over time and was influenced by the length of time users spent on the forum.

**Conclusions:**

Trust is a process that changes over time and which is influenced by structural features of the forum, as well as informal but collectively understood relational interactions among forum users. The study provides a better understanding of how the intersecting structural, relational, and temporal aspects that support the development of trust facilitate sharing in online environments. These findings will help organizations developing online health forums.

## Introduction

Online health forums provide peer support relating to a variety of medical conditions, including long-term illnesses, acute health problems, and life-threatening and terminal illnesses. Previous studies show that users value forums as a way of accessing emotional and informational support from people who are going through or have already undergone similar experiences [1-3]. Advice and support are solicited and provided by users sharing personal and sometimes very intimate experiences, and trust is therefore regarded as a necessary component in facilitating the sharing of peer-to-peer support [4,5]. However, less is known about the processes by which trust forms in such communities [6]. In this paper, we address this gap in knowledge by exploring how trust develops among users of an online forum for people with breast cancer.

With the advent and increased usage of online platforms including social media, forums, and discussion boards, the disclosure of personal information in online social spaces has become commonplace. Consequently, researchers have become interested in the phenomenon of trust in online environments. Some research has investigated trust in the system or structure, whereby the technological affordances of the platform (for instance, Facebook or a specific discussion board) are deemed more or less trustworthy, thus persuading or dissuading users from sharing their information [7,8]. Other studies have explored the similarities and differences between assessing trustworthiness in online and face-to-face social interactions [9] and investigated how people judge the trustworthiness of information online [10].

Trust is particularly significant within the context of online health forums, given the sensitive nature of the content that is shared and the extreme circumstances that individuals are facing. Studies have explored different aspects of trust in informing people’s decisions to share their personal experiences in such spaces. These include how technical affordances and the presence of moderators affect user inclinations to trust sites [11], the influence that individual personality traits and risk beliefs have on their inclination to disclose health information online [12], and the interpersonal nature of trust in online health forums, focusing on how user perceptions of relationships with other users affect whether and how they trust each other [5,13]. These studies have tended to regard trust either as a variable that can influence sharing or a behavioral outcome between and among forum users. Such approaches have been criticized for conceptualizing trust as a fixed and stable phenomenon, rather than taking into account its processual and relational nature, whereby trust emerges and develops over time and through changing interpersonal relationships [14].

A small number of studies have adopted a processual approach to conceptualizing trust, which is potentially more helpful in understanding how trust develops among forum users. Such research has identified that trust develops in separate stages or processes according to the trajectories of forum users and their changing relationships with other forum users. Radin [15], for example, identified 3 stages of trust on a breast cancer forum, in which users moved from lurking, to self-disclosure, to initiating virtual and face-to-face visits with other users. However, Radin used social capital theory to argue that people self-disclose by weighing up the potential risks and benefits of sharing about themselves which, we argue, implies a rational decision-making process that does not accurately account for user actions. Radin’s research also did not explain how users assess each other’s trustworthiness. Fan et al [16] focused on relational dimensions of trust to argue that users decide whether or not to trust each other based on the credibility of the information they post and their characteristics as posters (eg, frequency of posts and similarity to other users) and that some experienced forum users become trusted because of their established reputation and relationship with other users.

These approaches are useful in showing the interplay between relational and temporal dimensions in trust formation, in which trust develops between and among users and within online communities over time. Focusing on these 2 dimensions alone, however, risks downplaying the influence of the structure (for example, the platform) within which these trusting interactions take place. Sociological studies have conceptualized trust as a multidimensional phenomenon that operates at intersecting structural, relational, and temporal levels, whereby individual decisions concerning trust are influenced by social relationships over time, within particular sociopolitical contexts [14,17]. Rather than trust being considered an outcome or function of human actions, such approaches foreground the processes, contexts, and relationships within which trust becomes relevant and how individuals negotiate decisions about trust. We argue that a theoretical approach that conceptualizes trust as processual and relational, rather than as a fixed variable, is necessary to understand how trust emerges and develops. The overall aim of this paper is to examine how trust develops and influences sharing among users of an online health discussion forum.

## Methods

### Study Design

This study formed part of a larger project called a "Shared Space and a Space for Sharing" [18], which investigated how and why people in a range of extreme circumstances share information online and the importance of trust and empathy in the process of sharing. In this paper, we focus on how people with life-threatening and terminal illnesses use online support forums to share information, emotions, and experiences.

To gain an in-depth understanding of how trust manifests on the forum and develops between users, we chose a case-study approach and conducted thematic analyses of forum posts and interviews with 14 forum users. While most research has used either forum posts or interviews, few studies have combined these datasets [15,16]. We argue that using both provides a better understanding of how trust operates on the forum and that each dataset informs the other. For instance, while trust was rarely explicitly mentioned on the forum and therefore difficult to see by analyzing the posts alone, interviewee reflections on how they assessed and demonstrated trustworthiness shed light on how trust is done on the forum.

### Study Setting

After seeking permission from the UK charity Breast Cancer Care (BCC) [19], we chose an online forum hosted by the organization for our case study. We selected this forum because it is one of the longest established Web-based forums, it is open access, allowing anyone to view the site and read the message boards, and the terms and conditions made it clear that the information may be used for research purposes. While anyone with Internet access can view the material, users must register and log in to post messages. At the time of the study, there were an estimated 200,000 registered users, although not all of these are active at any one time (BCC, personal communication). Most users are women living with breast cancer. Other users include men with breast cancer, breast cancer survivors, and relatives and friends of those with the disease. The site is moderated by staff at BCC, whose responsibilities include ensuring that users do not disclose personal information that would reveal the identities of themselves or others, removing spam content, and intervening when necessary to manage conflict among users. The forum is organized into sections (eg, going through treatment), boards (eg chemotherapy, surgery), and threads (particular topics or queries initiated by users) within the boards. This description of the forum is how it was organized at the time of the data collection (2015). Since then, there have been a number of changes to the site, including the sections that are present in the forum [19].

### Discussion Forum

Purposive sampling was used to identify a range of discussion boards that would provide suitable diversity of the topics discussed. In 2015, we collected 233 archived threads across a range of years (2006-2014) from 10 different boards on the BCC forum (see Table 1 for details). The variation in dates reflects the fact that different boards were created over time. For each board, we started with the first post made to that space and collected subsequent posts until we had reached our sample quota. This was a pragmatic decision designed to sample consistently across different boards. The forum data were analyzed thematically according to the approach detailed by Braun and Clarke [20]. ML, PB, and JE familiarized themselves with the data in the sampled threads and noted initial ideas independently (phase 1). These were then discussed to compare the initial ideas emerging from the data and to enhance interresearcher reliability. ML then selected 15 threads from 9 of the boards (135 threads in total) for further in-depth analysis and coding (phase 2) (see Table 1). These threads were selected according to which were most pertinent to the project’s interests in the concepts of sharing, trust, and empathy. ML then coded the messages using NVivo 10 (QSR International), and a third of the messages were coded by PB and compared for analytical rigor. Following discussion and agreement between ML and PB, ML then grouped the codes into overarching themes (phase 3). These were then reviewed (phase 4) and refined by rereading the data extracts to ensure a close fit between the data and our conceptual interpretation of them (phase 5).

### Interviews

Interview participants were recruited purposefully via a message posted on the BCC forum, explaining the purpose of the project and inviting potential participants to contact the project team. Inclusion criteria were that participants be at least 18 years of age, a user of the BCC forum, and either have a diagnosis of breast cancer themselves or be a relative or friend of someone with the condition. Thus, our final sample was self-selecting and was comprised of users who proactively responded to the recruitment message. While it is possible that one or more of the interviewees may have written forum posts that we analyzed as part of our sample, we deliberately did not seek this information. As it was not necessary to analyze their forum activity to achieve the project aims, we decided not to ask interviewees to reveal their forum user ID as this would have compromised their online anonymity.

**Table 1:** Details of forum posts included in the analyses.

			Phase 1 analysis	Phase 2 analysis
Name of section	Name of board	Dates of posts	Number of threads	Number of threads
Going through treatment	Chemo monthly threads board	2013	1	
Welcome to the forum	New members board	2012-2013	25	15
Have I got breast cancer?	Appointments and waiting for test results board	2007-2008	25	15
I am recently diagnosed	Diagnosed with breast cancer board	2007	25	15
Going through treatment	Surgery board	2007	25	15
Living with and beyond breast cancer	Sex and relationships board	2012-2014	22	15
I have secondary breast cancer	End of life board	2009-2010	25	15
Supporting someone with breast cancer	Family, partners, and friends board	2006-2007	25	15
Talk to people like me	Younger women and families board	2007	25	15
Talk to people like me	Men’s board	2006	35	15

**Table 2:** Details of interview participants (all interviewees were women).

Name of participant (pseudonym)	Age range	Date of diagnosis	Type of interview
Anne	50-59	December 2014	Face-to-face
Beth	40-49	March 2012	Face-to-face
Christine	50-59	March 2014	Skype
Danielle	40-49	January 2015	Face-to-face
Eleanor	50-59	October 2012	Face-to-face
Frances	50-59	January 2014	Telephone
Gayle	50-59	June 2013	Face-to-face
Hazel	60-69	2012	Face-to-face
Isobel	40-49	January 2014	Telephone
Janice	60-69	March 2014	Telephone
Kathryn	50-59	December 2014^a^	Telephone
Libby	40-49	August 2014	Telephone
Nancy	40-49	December 2014	Telephone
Olivia	50-59	February 2010	Face-to-face

^a^Original diagnosis in 1992.

Interviews were conducted by JE with 14 forum users and took place between April and June 2015: 7 were conducted in person, 6 were by phone, and 1 was by Skype. Face-to-face interviews were conducted in a venue chosen by the interviewee. Table 2 presents the characteristics of the sample of interviewees, all of whom were women who had been diagnosed with breast cancer. All but one of the women were white British, with one white European. The mean age of participants was 52 years (range 40-67 [SD 8.0] years). The interviews were semistructured as we used a flexible schedule which guided the interview toward issues relevant to our study but allowed participants to introduce new topics. If a participant raised an issue of particular interest, this was incorporated into the schedule. Questions related to participant use of the BCC forum, their experiences of sharing online, their relationships with other forum users, and their experiences of and attitudes toward trust and empathy on the forum.

The shortest interview lasted 49 minutes and the longest was 2 hours and 30 minutes; the mean duration was 1 hour and 7 minutes. All interviews were audiorecorded and transcribed verbatim. Transcripts were analyzed thematically by ML following the 5-phase process described above [20], and interpretations and themes were discussed and agreed with PB and JE.

### Analyzing Both Datasets

Once both datasets had been analyzed in turn, we analyzed them together in order to triangulate our analysis and arrive at a better understanding of how trust operated on the forum. Combining the datasets in this way helped to shed light on themes emerging from the analysis of the forum threads that we had not initially associated with trust. For instance, one of the key themes which emerged from analysis of the forum posts was humor. While not immediately seeing the relevance of this to trust, analysis of the interview transcripts revealed that one way in which participants presented themselves and perceived others as trustworthy users was by including amusing stories in their posts. Consequently, combining datasets revealed aspects of the forum data that had hitherto been invisible to us. It was at this stage of analyzing both datasets that we developed our conceptual framework of the structural, relational, and temporal dimensions of trust, as we found this a useful approach to understanding how the themes from both datasets related to trust and also to each other.

### Ethical Considerations

The data collected from the online forum were publicly available, and under the terms of use of the BCC forum, users were informed that material posted was publicly accessible and so collecting informed consent from individual users was not necessary. This complied with the recommendations of the Research Ethics Committee. In order to preserve user anonymity, we deleted usernames and other identifying features (eg, names of organizations or other individuals). In accordance with the Research Ethics Committee’s requirements, we have reworded quotations in instances where it might be possible to trace a user by inputting the quotation into an Internet search engine. In such cases, care has been taken to protect the anonymity of the user while retaining the meaning and nuance of the original message.

Interviewees received information sheets about the study prior to providing informed consent and were informed that they could leave the project at any time. Although the purpose of the interviews was to ask participants to discuss their experience of the forums rather than their experience of the illness per se, we wanted to give interviewees the opportunity to talk about the illness as a way of contextualizing their forum use. We were therefore mindful that some participants might become distressed and developed a protocol with BCC of what to do in such instances; however, this was not required during any of the interviews.

Ethical approval was granted by the University of Sheffield Research Ethics Committee (analysis of forum posts; application 001955) and UK Ministry of Defence Research Ethics Committee (interviews; application 614/MODREC/14).

## Results

### Overview

Following our analyses, we conceptualized trust as operating in 3 different dimensions: structural, relational, and temporal. By structural, we mean how the affordances (the technological design of the forum which allows or prevents users from taking certain actions) and formal rules of the site affected trust. Relational refers to how trust was necessarily experienced in interactions with others; that is, it emerged within relationships and was a social rather than an individual phenomenon. The temporal dimension relates to how trust changed over time and was influenced by the length of time users had spent on the forum. These 3 dimensions intersect with each other and do not operate in isolation. For example, the trust which users had in the forum’s structure affected the extent to which they trusted and related to other users. Further, the more time the users spent on the forum, the more they got to know each other, meaning that the relational and temporal dimensions are inseparable. While for analytical purposes we separate the dimensions into 3 different themes, we draw out instances where they influence each other. As we triangulated analysis of interviews and forum posts, we intersperse both sets of data in the following sections. Anonymized quotations from interviews are indicated by a pseudonym and age group following the quote in square brackets; quotations from the forum are presented without usernames and followed by [forum post].

### Structural

Although the forum is accessible to anyone with Internet access, users must register before they can post messages. New users have their first 3 messages premoderated before their posts automatically go live on the forum. This enables moderators to identify and block spam accounts or other nongenuine users. Spam content is also screened out automatically by the forum’s filter system. Additionally, moderators remind new users that the forum is public and strongly advise them not to use their real names as usernames or to provide any other identifying information. Thereafter, moderators typically keep their interventions to a minimum, but these initial security measures were perceived by participants as contributing to the safety of the forum and inclined them to trust other users, as they believed them to be genuine.

I do trust the site, yes...when you’re completing, you know, the name that you’re going to use on there, it’s very much that you only put as much information as you want...so I feel we’re quite secure in that way, um, and the things that people are putting, um, you trust what they say.

Frances, 50-59 years

While few posts explicitly mentioned trust in the forum, users often assured new posters that they found the site supportive and helpful.

...do share any worries or problems in the forum—all of us understand and there is always someone who you can rely on to help you...

forum post

New members of the forum are also given an initial message of support from a forum moderator.

...I am sure you will get plenty of useful advice and information from other people on the forum. You are also welcome to contact our Freephone helpline on 0808 800 6000—you can talk to someone confidentially about how you are feeling.

forum post

This welcoming message from a moderator, in addition to welcome messages from other users of the forum, gave new members confidence in the forum and helped them to develop trust in the online community.

Within minutes I got loads of responses that were really reassuring and I thought, oh great, that’s really helpful...

Anne, 50-59 years

This structural aspect of the forum was important at a time when the new member had made their first post and emphasizes the importance of temporality, in that getting a swift reply from forum users in response to their first post was reassuring. It also highlights the relational aspects of helping the new member develop trust and relationships within the community.

However, despite the forum’s formal procedures and guidance to users, not all interviewees realized that the forum was public, and one participant described being upset when an offline acquaintance discovered some personal information about her from reading the forum.

Well, up until I found out about, you know, the incident, I felt sort of 99.9 percent safe but now obviously I don’t because I realize that people can come in and identify you...so it’s not as safe as I thought it was.

Christine, 50-59 years

While overall the forum was regarded as a safe space in which participants felt they could trust each other, the incident described above shows that it was not able to guarantee participant anonymity completely and this could influence the trust which participants had in the site and how they shared information about themselves. In this instance, it appeared that the user had placed too much trust in the site, from her misunderstanding of how open the forum was, and had shared more information than she would otherwise have done.

The information shared on the forum was also generally trusted because users believed that the BCC moderators would act to ensure that no false or misleading advice was posted. In the context of a cancer diagnosis, trust in the informational content of the forum was perceived as being particularly important, as users may have been more desperate to try different treatments.

The trust on the information that you can pick up is different, um, because...around cancer there is a lot of um—well, it’s a scary thing. It’s life and death and people um, when their backs are to the wall will try anything and everything. You know, it’s sort of drinking your own piss type territory. So, any, um, advice, I think has to be, um, looked at quite carefully and I think the...moderators actually monitor that so I think most of what is on there—all of what is on there is factual, informative or um, um, it’s been moderated; maybe been taken down...[the moderators] might say “this isn’t helpful” and that is a very good thing because it does, um, clean up to some extent the site and it makes it more trustworthy.

Hazel, 60-69 years

As the quote implies, because the forum was for people with cancer it meant that being able to trust the content posted was particularly important. The consequences of acting on misinformation may have resulted in serious negative health effects or using products that were not supported by an appropriate evidence base.

Forum users also provided guidance on the extent to which information on the Web could be trusted.

...Be very careful with what you read on the Web. Much of this is out of date, anything that’s over a year old is out of date...

forum post

This kind of advice, coupled with the presence of moderators and the feedback from other forum users, helped users to develop trust in the forum.

The availability of the forum 24 hours a day meant that someone could post a message whenever they needed to share their feelings with others (eg, in the early hours of the morning when they felt anxious and could not sleep). It was also possible that there might be others online at that time who were able to comfort and provide some reassurance, helping users to trust that this could be a reliable source of support throughout the day and night.

My first post was...like two in the morning when I couldn’t sleep and I was like...okay, can’t sleep, rollercoaster whatever, anybody else feeling this? Is this normal? I’m crying half the night, can’t sleep...and you kind of—somebody’s up there all the time regardless (laughing) of what time of the night or day it is and...somebody came back saying yeah, it’s normal, hear you, been there, it will get better. It’s kind of that reassurance...

Danielle, 40-49 years

### Relational

The site facilitated peer-support via the sharing of personal experiences, emotions, and information; it was therefore vital that forum users trusted each other. While trust was not often explicitly mentioned on the forum, interview participants indicated the ways in which they assessed the trustworthiness of other users and also demonstrated their own trustworthiness. In an online rather than face-to-face medium, in which they were unable to see gestures or facial expressions, trust occurred in the following ways: using an appropriate tone, being reciprocal, not claiming expertise that users did not have, and seeking or demonstrating similarity with other users.

#### Appropriate Tone

Using an appropriate tone in posts was regarded by participants as a key way in which they could demonstrate or assess trustworthiness. Inappropriate posts included those perceived as ranting or being overly negative. Such users were regarded as being too engrossed in their own concerns rather than responding to and supporting other users.

The other thing is it’s not okay to be a negative person on the website...Everyone’s allowed a one-off meltdown so long as you get over it the next day or the day after. You are not allowed to be permanently miserable because if you are, you get pretty much ignored...So, the whole trust thing, “Can I trust you with personal information?” “Yeah.” “Can I trust you to want to interact with me?” “Yeah, only if I do it in the right way” (laughs).

Anne, 50-59 years

Although it was clear from the interviews that it was acceptable for a person to express negative feelings on occasions, it was not acceptable to be negative continually. To be trusted and for other users to respond to their posts, individuals were expected to be aware of and respond to the needs and feelings of others. This was also revealed in many instances of posts in which users apologized for ranting.

Sorry for ranting but I want to know what one out of 3 sentinel lymph nodes having cancer means?

forum post

Sorry to witter on but I really want to describe the context.

forum post

These demonstrate user awareness of not appearing overly preoccupied with their own situations and to be mindful of the effect this might have on others. As demonstrated in the quote from Anne, being perpetually negative was not tolerated and hindered trust, although occasional rants were acceptable and could foster trust.

Humor was also frequently used in posts as a way of conveying the right tone, even in very serious circumstances. Interviewees explained the value of humor in lightening what could otherwise be a very dark space and demonstrating trustworthiness.

...for me there was a sort of balance, but even if you were having a really shit week, you always said something hilarious or something...There’s always something stupid and ridiculous that happens.

Olivia, 50-59 years

...I tend to turn to humor a little bit if I can because if you can’t laugh, what are you going to do? (laughs).

Libby, 40-49 years

The following posts were typical of the humor presented on the forum.

Having been diagnosed on Valentine’s Day (the consultant didn’t even write it in a card for me!) for breast cancer, the plastic surgeon recommended a skin saving mastectomy/reconstruction.

forum post

Seriously it was much better than I was expecting. Obviously, no one wants to be in hospital but I felt relieved that the cancer was out. My boob and I were no longer friends after diagnosis so I wasn't that sorry to see it go in the end!

forum post

As well as humorous language, users frequently included emojis in their posts to help them convey appropriate emotions.

You’ve always got to have a smiley...smileys or emoticons are brilliant and now you can get the moving ones and all sorts...if you’re annoyed you can have a big, red, angry face or whatever but...I mean, I was always a smiley user. If you read my posts there’s probably a smile or something stuck on it...they weren’t very good ones but...I think you can get some of it across by—by your emoticons or smileys...

Beth, 40-49 years

Language was crucial in demonstrating to each other that users knew the informal rules of the forum: that it was acceptable to share negative emotions such as anger or distress as long as this was done in an acceptable way, meaning that they would appear trustworthy. Conversely, forum users who were perceived as being overly negative in their posts or who did not moderate their comments either with humor or an explanation were mistrusted by other forum users because they transgressed the accepted norms.

#### Reciprocity

Trust was also influenced by the extent to which users displayed reciprocity. As a peer support forum, it was imperative that users provided as well as received support. Reciprocity could be demonstrated through language, and, relating to the previous theme, users had to convey a reciprocal tone in order to be trusted.

I think trust as well, for me, um, when people would go on and they’d just been diagnosed and they were ranting and all the rest of it and then they went on and on and on being me, me, me members of the forum and in the end I just wouldn’t respond to them anymore because they never responded to anybody else. They never contributed.

Olivia, 50-59 years

Reciprocity could also be demonstrated through behavior such as explicitly responding to others or being a frequent user of the forum. Such reciprocal behavior conveyed that the person was trustworthy which, in turn, facilitated the sharing of support and the development of a sense of belonging to the forum.

The people I suppose that I trust are the ones that are there regularly first off...I suppose the people that actually try and answer your questions...it is just about “Do I feel this person understands what I’m trying to say and is trying to help me?” and if they are, then I do—try and do the same for them, and other people it’s a bit more of a soapbox. It’s a bit more, you know, “I feel terrible and you all should run around and help me,” but actually, you’ve never bothered to post an answer to anybody else’s thing or whatever and you just think, well, you know. Again, I might answer the first few but eventually I’m probably not going to bother because it’s all about sharing and trying to help each other.

Anne, 50-59 years

I thought that frequency was really good to develop trust, that if you’re on there fairly frequently people kind of trust that you’re serious, that you—you know, that you care about them, that you—you’re part of the forum; you kind of belong if you like.

Olivia, 50-59 years

Reciprocity was also a temporal as well as a relational characteristic as users applied their knowledge of each other’s past actions to help them predict future actions, thus influencing their immediate decisions on the forum regarding trust. Reciprocity and feelings of regard for other users were evident in the frequent posts which explicitly thanked others for their support and offered well wishes.

Thank you once again to everyone who responded, it is so comforting to know that people out there who have never met me care enough to spend their time carefully thinking through and writing replies. If anyone needs advice from me about something they are going through, or about to go through then I will be very pleased to help.

forum post

Thanks everyone for replying. I feel a lot better now. This is a wonderful forum. I’d been crying so much but you have all made me feel better. Good luck with all your treatments.

forum post

Reciprocity therefore indicated that users were good forum users; they were concerned for each other and not just for themselves, which in turn helped to foster trust on the forum.

#### Not Claiming Expertise You Do Not Have

Just as forum users perceived that moderators helped to facilitate trust by monitoring and removing misinformation, users also assessed each other’s trustworthiness according to their claims to expertise. Interviewees reported that they were inclined to distrust users who appeared to provide medical information without the caveat that they could only speak from personal experience.

I worry about people who give answers on things they’re not qualified to comment on. So, I think anyone can advise anybody else of what they think but (unclear) don’t present it as, you know, fact when it...isn’t...particularly when you’re talking about life-threatening illnesses...you know, clearly everyone’s head is going to be in a mess, so...that’s the absolute worst time to be presenting yourself as an expert in anything. Qualify it, you know, if you’re going to say it and I am astonished at the number of people—“Oh, I’d never do this” or “You must always do that.” I just think “How do you know?” (laughs).

Anne, 50-59 years

Another interviewee reported that if she read a post containing medical advice or information that she believed to be incorrect, she intervened to try and present a fuller account out of concern that other users might trust the content

...she was telling these people on the site not to eat sugar because sugar feeds the cancer, and I know...what she was sort of thinking...but that would have put the fear of God into a lot of people...I don’t always trust what I read but that’s usually—it’s a medical thing and I think, well I know better than that but um, it’s hard when there’s other people that wouldn’t know and then obviously read things like that...my concern is that, you know, there are people on there that—if they’re trusting everything they’re told, it can be a little bit of a scary place, um, because, you know...they then suddenly think oh God, I’m not going to eat any more sugar or I’ve got to go and do this...quite often if I see a comment like that I will comment and direct them to a site that will explain to them what that person’s been trying to say with regards to, you know, the cancer needing energy and what have you...

Frances, 50-59 years

Frances’ interventions also suggest a feeling of responsibility toward other forum users which could engender trust, both in making people aware that not all information should be taken at face value but also directing them to websites containing more reliable information.

Forum posts often contained medical information in which users emphasized that they could only speak from their own experience.

I can tell you about my decisions about hormone therapy but need to emphasize that this was my personal decision.

forum post

The four-week gap is very long (in my opinion).

forum post

In this way, forum users framed their advice as being derived from their own experiential knowledge rather than their being an expert.

#### Similarity of Other Users

There was also evidence to suggest that trust was influenced by the extent to which users shared similar characteristics or situations, with some interviewees saying that they were more inclined to trust other users who were of a similar age or who had a comparable family situation

Interviewee: Interestingly...when I’ve looked at the people that I reply to and speak to, generally on the forum, they’re often very similar...So, when I look at—if I look and analyze or think about, um, the people that I liaise with, um, maybe on a weekly basis, that they seem to be similar people, you know.

Interviewer: And that helps you to trust? Is that what you’re—? Yeah.

Interviewee: Yeah, yeah. I think so, yeah.

Eleanor, 50-59 years

There were frequent references in user posts to characteristics such as their age and how many children they had, and the insight provided by the quote above suggests that inclusion of these details may have informed user decisions on who to trust (ie, because they were in a similar situation, not only with respect to being affected by breast cancer but also in terms of their personal circumstances). Examples of posts in which users emphasized their similarity to each other follow.

What time is your appointment tomorrow? I’ll be thinking of you—our situations seem similar—I have a 7-year-old and a 2-year-old.

forum post

I was reading your post and felt the need to reply. I lost my beloved mum just over a year ago after her breast cancer spread to her liver and stomach. Like you I am an only child and was very close to my mother.

forum post

The inclusion of such details may help to place users and make them appear more relatable and familiar to each other. This was something that seemed important in an online setting, where users are more limited in their ability to assess the extent to which other users are their kind of people than in face-to-face support groups.

### Temporal

The BCC forum included a range of users, from those who were new to the site following a recent diagnosis through to users who had been using the site for several years. The ways in which users experienced and perceived trust did not necessarily remain constant during the time they spent on the forum but changed over the course of their illness and as they gained experience of using the forum and getting to know the other users.

#### Lurking or Deciding to Post

Interviewees had different experiences of and perspectives on initially joining the forum and deciding if and when to start posting. Some users joined the forum and started posting relatively quickly after diagnosis. For Janice (60-69 years), the extreme circumstance of receiving a cancer diagnosis and undergoing chemotherapy provoked the formation of trust among users “because chemo is such an intense experience that that trust is actually forged, that sort of initial bit.” Another interviewee described herself as “an innately trusting person, so my default is I trust somebody until they prove otherwise...Um, I think the developing of trust—I think you just test it out don’t you? You put something out there and you see what people say back…” [Olivia, 50-59 years]. In such cases, the trust required to start posting on the forum developed quite quickly.

Other users lurked for a period of time in order to get an idea of what the forum was like and to judge “Are these my kind of people?” [Anne, 50-59 years].

In their initial posts, forum users often mentioned that they had watched the forum for a while before deciding to post.

This is my first post as I have learned a lot from reading your posts but have always been too nervous to join in.

forum post

I’m excited to say this is my very first post! Hope you don't mind me joining in but I've been lurking and reading everyone else’s messages for about 11 months now and thought it was about time I joined in.

forum post

Making the initial decision to trust the forum and its users enough for new users to post was therefore influenced by the nature of the reason for accessing the forum (ie, breast cancer) and users’ natural inclination or disinclination to trust people.

Interestingly, users who posted did not always consider or were not concerned that their posts might be read by lurkers or anyone else who accessed the forum. The trust they developed was in other people who posted on the forum, but they did not appear to take account of other people who were not visible.

#### Developing Trust Over Time

The trust which users had in the forum and toward other users changed over time, and participants spoke of trust growing as they got to know other users better.

I do trust the women that I’m in the group with. Um, I suppose that builds as time goes on. Um, you don’t know them to begin with and then, by now, you know, six months down the line you know them and we know each other on Facebook as well now. So, we know each other’s real names (laughs) for instance. Um, so I do trust them...I think just, um, because I do only more or less post in the one thread with the same people, it’s—you know, it becomes familiar with this set of people and—yeah, you’re chatting about something with somebody that you know essentially.

Libby, 40-49 years

This suggests that users did not necessarily develop trust in the forum as a whole or with everyone but used particular sections or threads or where the same people tend to post and so become familiar with each other. This is an example of how the forum’s structure intersected with the relational and temporal dimensions of trust formation, particularly in providing separate spaces, within which users got to know people and develop relationships over time.

Forum posts often included details of users’ everyday lives, such as their holidays or hobbies, and these—as well as the information about their treatment and health condition—may have helped users to get to know each other better. This may have helped the development of trust.

I have to like someone before I’ll trust them and I have to know them quite well really, yeah...going back to how you perceive people by how they write, which you have to—and you bear in mind that...you start speaking to somebody from, say—for almost six—daily for six months, which you probably wouldn’t speak to your best friend daily for six months. So, you learn an awful lot about people each day and...you just have a conversation really and you...get a good um, idea of what people are like, unless they’re very good at inventing a story about themselves, which I’m sure some people could, but on this particular kind of thread you wouldn’t—you wouldn’t make things up really.

Beth, 40-49 years

This also reiterates the importance of users demonstrating their trustworthiness through their writing and how this facilitated trusting relationships. Forum posts would sometimes allude to users knowing each other based on previous posts.

You are definitely right David. I’ve read some of your messages before and know that you are a warm caring person who cherishes his wife and family and is not ashamed to tell everyone.

forum post

In this way, trusting relationships developed over time.

## Discussion

### Principal Findings

The aim of this paper was to examine how trust develops and influences sharing among users of an online health discussion forum. In this paper, trust as a processual practice emerges as a complex concept involving a number of elements. Interviewee accounts in particular suggested that trust plays an important role in how people share information, experiences, and emotions in online health forums. In our study, we explored how trust manifested on the BCC forum in 3 main intersecting dimensions: structural, relational, and temporal. The structural affordances of the forum (eg, the presence of moderators and security features) inclined users to trust the site as a safe space where they could share personal details. Within the forum’s structure, users conveyed and assessed trustworthiness relationally, that is, through their interactions with other users. Relational trust involved using appropriate language and tone in forum posts, behaving reciprocally, not falsely claiming expertise, and seeking similarities with other users. Trust was also necessarily a process that developed over time, from the initial decision by users to join the forum to ongoing changes in how users related to each other as they got to know one other.

In addition to confirming findings in previous studies, our paper makes 2 new key contributions to the literature on trust in online health forums. First, by triangulating analyses of forum posts and interviews with users, our research reveals the characteristics involved in assessing and conveying trust such as not ranting and deploying humor. Interviewees spoke explicitly of how these were instrumental in assessing and negotiating trust, and this was also evident in forum posts. While previous studies have identified that tone, humor and the temporal trajectory of forum users facilitate sharing on online breast cancer forums, they do not show explicitly, as we do, how these characteristics are related to trust [21-23]. Additionally, while studies of face-to-face interactions between health professionals and patients with cancer have identified that humor relates to trust, different conclusions have been made as to whether trust must be present before humor is used or if it emerges as a result of humor [24,25]. Our research supports arguments that humor contributes to the creation of trust and does not just result from it. Our findings also support research that suggests that people are more likely to perceive online information as credible when it is posted by users who are judged to have similar characteristics to themselves [26].

Second, while previous studies have considered how, separately, structural [11], relational [16], and temporal [15] dimensions influence trust on online health forums, our study demonstrates that these dimensions intersect and cannot be adequately understood in isolation from each other. Here we draw on sociological theories of intersectionality, which emphasize how analysis of empirical data is enhanced by considering how different dimensions interact with each other to shape how a particular phenomenon, experience, or identity is manifested [27]. We argue that through intersecting with each other, the structural, relational, and temporal dimensions become more than their distinctive components, revealing that trust is processual and fluid rather than a fixed, unchanging variable. The structural affordances of the online spaces within the forum intersected with relational and temporal dimensions of trust by enabling people to get to know and trust each other by responding empathetically over a period of time. In addition to the structure of the forum allowing relationships to develop over time, this also allowed users to communicate with each other 24 hours a day. Consequently, the forum’s structure facilitated relational interactions between users at times when users’ family members and friends may have been unavailable to provide support. The organization and moderation of the BCC forum was relatively light-touch and, after new users registered and had their initial posts checked, the forum’s structure may have been largely invisible to users apart from when moderators occasionally intervened. We suggest that this reveals the permissive rather than restrictive nature of the structure and is not indicative of the forum’s insignificance as an influence on sharing practices. We find sociological conceptualizations of trust useful in highlighting these intersecting dimensions. For instance, as Brownlie and Howson [17] suggested in their study of trust in the context of MMR vaccines, “leaps of faith cannot be understood outside interactions and relationships nor isolated from the systems or institutions within which these unfold.” Similarly, Khodyakov [14] has argued that conceptualizing trust as a process requires understanding temporal characteristics of trust and how they influence trusting relationships within systems or structures. Our 3-dimensional theoretical framework helps us to make sense of the processual nature of trust on such forums. In doing so it contributes to the gap identified in a recent meta-synthesis of qualitative studies of online communities for people with long-term health conditions, which concluded that while trust and reciprocity do exist in such sites, “far less is known about the process that facilitates them” [6].

### Practical Implications of our Study

Our study has practical implications for how organizations that create and maintain online peer-support forums can help to make these trusted spaces where users feel able to share information and experiences. A growing literature suggests that organizations that manage online communities can cultivate trust through particular design features or management practices [16,28]. Our study adds to this knowledge through highlighting the importance of having in place appropriate structural aspects of forums—for example, a registration process and formal moderation. Moderators can encourage trusting relationships between users by removing offensive, inaccurate, or inappropriate posts. The structural aspects of forums help users to trust the online environment within which they elicit information from others about their condition, treatment, side effects, etc. This also helps people feel confident about sharing their own information, experiences, and emotions, and organizations that develop online discussion forums for patients and carers should ensure that these structural aspects are in place to help foster trust in the forum.

On the one hand, our findings show ways in which structural features built into forums can influence how users trust each other in a top-down approach. On the other hand, our research demonstrates how trust is also a bottom-up phenomenon that emerges out of ongoing relationships and informal rules. This suggests that structural elements may not influence trusting relationships in a simple cause-and-effect relationship but may be hard to predict and dependent on relational and temporal aspects. Our more nuanced findings indicate that future collaborative work involving organizations and researchers is needed to explore possibilities for forum design and management that are based on a more processual understanding of the agentic and emergent nature of user relationships. Ultimately, the interdependence of the 3 dimensions in the formation of trust suggests that organizations developing online discussion forums need to be aware of the importance of these as intersecting components for developing trust among users of forums.

Our study also has implications for clinical practice. People increasingly turn to online health forums on receiving a diagnosis, and while recent research suggests much of the information shared on such sites is of good quality [29], our findings can be useful for clinicians advising patients on what characteristics (eg, tone, claims to expertise by users) to look for before deciding to participate in online forums. Through using health forums, patients can become better informed about their condition and play a more active role in the decision-making process.

### Strengths and Limitations of Our Study

The strengths of this study lie in its in-depth exploration of one online peer-support group and in combining analyses of forum posts and interviews with forum users. Using both datasets together enabled us to strengthen our analysis and the validity of our claims. We demonstrate instances where analysis of the interview transcripts helped us better understand themes present in the forum posts and vice versa. This approach is useful in analyzing the inner workings of online forums, where it would be difficult to understand user intentions and decisions from their forum posts alone. We argue that using these complementary approaches can be particularly beneficial in researching intangible phenomena such as trust, which are not necessarily explicitly discussed or made visible on forums [30].

Our study is limited to a single case study that concerns one health condition, breast cancer. The forum is predominantly used by women and all of the interviewees were women. All interview participants self-selected, and this may have introduced elements of bias. For instance, forum users with strong opinions toward the forum may have been more likely to respond to the recruitment message than others. It is likely that trust manifests differently on other forums used by people with different illnesses and of different demographic groups [31]. In particular, we are aware that experiences of having breast cancer are shaped by distinct sociocultural discourses, and therefore our findings must be interpreted within this broader context. Other conditions may have certain characteristics that shape collective identities, influencing the content and nature of online interactions. We hope to explore this in future research. However, we suggest that the theoretical framework used to interpret our findings here has wider applicability to other online health forums where trust is of significance.

### Implications for Further Research

Further research could examine how trust operates in relation to what is shared in the online environment—for example, whether it is factual knowledge and how important this is to personal health and well-being and the personal and experiential nature of information and emotions that are shared. It is important to understand the extent to which the person sharing these different types of information is placing trust in the forum users as well as the trust that people place in the information. What is clear from our research is that trust and how it appears in online environments is highly contextual and content specific, and so further research is needed to explore how the process of trust may change on different forums with different demographic groups. Future research could also examine in greater detail the role of moderators in shaping trust in online spaces. Finally, new technologies and platforms are likely to change how trust manifests, so researchers should be responsive to technological change and how this might affect trust and sharing online.

### Conclusion

This study contributes new knowledge to the underresearched area of how trust forms and develops on online health forums. Our findings show that the development of trust is a process which is influenced by structural features of the forum, and informal but collectively understood relational interactions between forum users. It is also apparent that trust changes over time. We suggest that this 3-dimensional framework of trust could be applied to other studies of trust in online health settings. Our findings are of value to organizations hosting online health forums that seek to develop a better understanding of what promotes trust and facilitates sharing in online environments.

## Abbreviations

BCC: Breast Cancer Care
